# Dyadic Effects of Attachment on Illness Acceptance in Patients with Breast Cancer and Spousal Caregivers: Sense of Coherence as a Mediator

**DOI:** 10.3390/jcm13216425

**Published:** 2024-10-26

**Authors:** Dariusz Krok, Ewa Telka, Marcin Moroń

**Affiliations:** 1 Institute of Psychology, University of Opole, 45-040 Opole, Poland; 2 Department of Radiotherapy, Maria Sklodowska-Curie National Research Institute of Oncology, Gliwice Branch, 44-101 Gliwice, Poland; etelka@io.gliwice.pl; 3 Institute of Psychology, University of Silesia in Katowice, 43-126 Katowice, Poland; marcin.moron@us.edu.pl

**Keywords:** actor–partner interdependence model, attachment, illness acceptance, sense of coherence, breast cancer patients

## Abstract

**Background:** Attachment styles have been found to play a significant role in adjustment to cancer. Couples often cope with breast cancer through an interdependent approach to the disease rather than just acting as individuals, and a sense of coherence is an important factor that influences these relationships. This study examined how attachment styles and sense of coherence impact illness acceptance in couples facing breast cancer within a dyadic perspective. **Methods:** Data were analyzed from 145 women with recently diagnosed breast cancer and their 145 partners, who attended clinic appointments related to medical treatment. They completed self-report measures of attachment, sense of coherence, and illness acceptance. **Results:** Higher secure attachment and low insecure attachment scores were associated with a higher sense of coherence and better illness acceptance both in women and partners. Results of actor–partner interdependence mediation models indicated that most associations between attachment styles and illness acceptance were mediated by sense of coherence within both intrapersonal (actor–actor) and interpersonal (actor–partner) effects. **Conclusions:** The interdependence in attachment and sense of coherence brought noticeable benefits to couples’ illness acceptance when facing breast cancer. In line with the salutogenic model, these relationships predominantly depended on the mediational function of comprehensibility, manageability, and meaningfulness, which determined cognitive and emotional reactions that influenced both patients’ and spouses’ acceptance of the disease.

## 1. Introduction

Breast cancer is one of the most common forms of malignant tumors affecting women worldwide [1]. In Poland, more than 22,000 women are diagnosed with breast cancer each year, of which 5–10% are directly diagnosed at an advanced stage [2]. The severe physical consequences of breast cancer (e.g., mastectomy, pain, chest scars, bodily dysfunction) greatly impact the quality of life of both women and their spousal caregivers. Examining the relationship between marital attachment processes and illness acceptance within the framework of the dyadic approach is therefore extremely important, both for scientific and therapeutic purposes.

### 1.1. Associations between Attachment and Illness Acceptance

In the context of family relationships, an important factor that determines the quality of marital life is attachment, mainly due to the psychological basis for the formation of attitudes of closeness, relationality, avoidance, and anxiety among spouses [3]. In particular, the quality and extent of attachment significantly influence marital emotional responses and the support shown in difficult life situations. According to Bowlby, attachment style represents how an individual seeks closeness and forms interpersonal relationships; attachment styles develop in infancy and often remain stable over time [4,5]. In adulthood, attachment styles are usually assessed per dimensions of anxiety and avoidance, defining how individuals communicate and respond emotionally. People with an anxious attachment style are inclined to express closeness and show excessive signs of concern, whereas people with an avoidant attachment style tend to be emotionally uncomfortable and less willing to show sensitive support [6]. Conversely, individuals with secure attachment (low anxiety and avoidance) are characterized by showing intimacy and interpersonal closeness. This indicates the different nature of attachment styles in relation to social communication characteristics.

In the context of marital relationships, several studies have found that attachment styles play a significant role in times of suffering in patients with cancer and their partner/spousal caregivers. In couples coping with lung cancer, partners’ anxious attachment (low levels of intimacy and closeness) was associated with patients’ worse adjustment in terms of detrimental cancer symptoms. Furthermore, avoidant attachment in both patients and their partners was associated with higher levels of symptom burden in patients (e.g., fatigue, uneven breathing, nausea) [7]. Research using actor–partner interdependence mediation models has shown that insecure attachment styles (anxious and avoidant attachment) were related to poorer physical well-being among couples coping with various types of cancer (stage II–IV breast, lung, colon, or rectal cancer). In addition, these associations were mediated by two communication behaviors, disclosure and holding back, which were employed to assess the above-mentioned relationships [8]. Higher anxious and avoidant attachment was related to a higher level of negative affect and negative approach behavior among both cancer patients undergoing chemotherapy and/or hormone treatment and their partners; higher avoidant attachment was also related to less positive affect [9]. Furthermore, patients and partners higher in anxious and avoidant attachment were characterized by poorer physical well-being; however, the effect of avoidant attachment was stronger for patients than partners.

These results are consistent with a recent meta-analysis conducted on female breast cancer patients, which revealed that anxiety and avoidance dimensions of insecure attachment were associated with lower quality of life and higher distress. More specifically, avoidant attachment in comparison with anxious attachment was more frequently and more strongly related to more negative outcomes, reflecting poorer psychological adjustment [10]. This suggests that cancer patients tend to be more likely to endorse avoidant attachment than anxious attachment. Other studies have also confirmed the important, but diverse, role played by attachment styles in the well-being of patients with cancer and their caregivers [11,12].

These findings indicate that insecure attachment is associated with negative physical and emotional indicators of well-being among cancer patients and their partners/spouses. Yet, despite growing empirical evidence, the underlying mechanisms responsible for the associations between attachment and well-being are not fully understood and require further investigation, especially in terms of actor–partner interdependence mediation models. To our knowledge, no study has examined the relationship between attachment styles and illness acceptance among couples coping with breast cancer. 

### 1.2. Sense of Coherence as a Potential Mediator

In times of serious illness, individuals are likely to co-regulate their attachment styles on a basis of psychological resources, which in turn can influence their well-being and illness acceptance [13,14]. Some empirical evidence indicates that the association between attachment styles and illness acceptance may be mediated by factors that augment individuals’ emotional responsiveness (secure vs. insecure attachment) and subsequently influence a reinterpretation of the disease. In a group of couples facing ovarian cancer, the relationship of both one’s own and one’s partner’s greater insecure attachment with social and functional quality of life was mediated by common dyadic coping [15]. Rather surprisingly, greater common dyadic coping experienced by one’s partner was related to one’s own lower quality of life, which indicates the exhausting character of this style of coping. Among patients with a cancerous brain tumor, perceived social support was found to mediate the associations between attachment anxiety and attachment avoidance and helpless/hopeless coping, suggesting that insecure attachment is related to social relatedness factors on a mediational basis per the differences in coping styles [16]. Emotional self-disclosure also mediated the relationship of attachment anxiety and attachment avoidance with quality of life among young breast cancer survivors, which indicated that attachment processes can play a significant role in breast cancer patients’ adaptation to the disease [17].

Given the important role of purpose- and meaning-related factors in the psychological functioning of cancer patients [18,19], it seems highly plausible to assume a mediating role for sense of coherence in the associations between attachment and illness acceptance. Perceiving one’s life and experienced illness in terms of comprehensibility, manageability, and meaningfulness—which comprise a sense of coherence—allows individuals to be more resilient to the stressors of daily life, maintain well-being, and improve their health [20,21]. Consequently, ill people can modify their attitude toward their illness and begin to perceive it in a more constructive and less burdensome way.

One study found that, in cancer patient–caregiver dyads, the caregiver’s sense of coherence mediated the relationship of the caregiver’s perceived social support with the patient’s acceptance of illness, and the patient’s sense of coherence mediated the relationship of both caregivers’ and patients’ perceived social support with the patients’ illness acceptance [22]. Sense of coherence was also a mediator in the associations between different forms of social support (i.e., perceived available support, actually received support, and protective buffering support) and illness acceptance among breast cancer patients [23]. In ethnically diverse groups of college students, sense of coherence mediated the relationship of attachment and college challenges with depressive symptoms [24]. These findings suggest that sense of coherence as an inner resource may serve as an underlying psychological factor in the associations between close emotional bonds and acceptance of breast cancer. However, research had not examined sense of coherence as a mediator in the relationship of attachment with illness acceptance within a dyadic approach among patients with breast cancer and their spouses.

The relationships proposed above appear possible within the context of Antonovsky’s salutogenic model that has been widely applied in health psychology [20,25]. According to the model, one’s physical and mental health are mainly determined by sense of coherence, understood as an internal resource which helps individuals mobilize both general and specific resistance resources. In the face of psychosocial and physical stressors (e.g., illness), it is used to manage stressful and challenging life situations, which, when resolved constructively, lead to improvements in a person’s mental and physical functioning. The model also posits that attachment processes derived from relationships with loving parents or supportive friends play a significant role in rendering the world comprehensible, meaningful, and manageable, perceptions which form the core of sense of coherence [25]. Thus, it seemed likely that sense of coherence would mediate the association of attachment with illness acceptance attitudes, especially taking into account its specific features. Previous research found that attachment increased sense of coherence, which, in turn, mediated its protective effect against depressive symptoms in a non-clinical sample of college students [26]. The current study built upon this research and simultaneously extended it by examining attachment, sense of coherence, and illness acceptance in a clinical group of cancer patients and their spouses within a dyadic model.

### 1.3. The Present Study

We aimed to examine whether sense of coherence mediates the association of different attachment styles with illness acceptance experienced in breast cancer dyads (women with breast cancer and their spouses). Moving forward, we refer to all non-patient partners as spouses to avoid confusion with statistical partner effects. Based on previous research [22,24,26], we hypothesized that patient and spouse secure attachment would be positively associated with their own sense of coherence and illness acceptance, whereas patient and spouse avoidant and anxious attachment would be negatively associated with their own sense of coherence and illness acceptance. Also, a person’s sense of coherence would mediate the effect of their own attachment on their own illness acceptance (i.e., the actor–actor effect). Finally, a person’s sense of coherence would mediate the effect of their own attachment on the illness acceptance of their partner (i.e., the actor–partner effect). To address the last two hypotheses, we tested the mediating role of sense of coherence in the actor–partner interdependence model (i.e., actor and partner effects of sense of coherence on illness acceptance).

## 2. Materials and Methods

### 2.1. Participants and Procedure

Patients (women) and their spouses were recruited from cancer centers in southern Poland during clinic appointments related to their treatment. Potential participants were identified from the cancer center’s medical records. The inclusion criteria were as follows: (a) women with stage I–III breast cancer diagnosed within 6 months; (b) women married (cohabiting) and living with a spouse; (c) minimum patient age of 18 years; and (d) patient was without serious mental or cognitive impairment. Exclusion criteria were as follows: (a) extremely adverse reactions to treatment; (b) severe mental disorders; (c) severe cognitive impairments; and (d) metastases to parts of the body other than the breast.

Eligible participants were approached by a research assistant at the cancer centers and invited to participate in this study. Couples who agreed to participate received detailed information about the study, a written informed consent form, and a questionnaire to complete. After receiving detailed information about the study and providing written informed consent, both patient and spouse received a questionnaire with the request for it to be completed on one’s own time. Completed questionnaires were returned at the clinic and then collected by a research assistant. 

Of 384 eligible respondents, 290 participated in this study (75.5%). The main reasons for not participating were lack of interest in the study (15.4%), unexpected medical circumstances (5.5%), and other reasons (3.6%). The study was approved by the Ethics Committee of the University of Opole (No. KOJBN 6/2022) and was anonymous. Participants were recruited on an informed and completely voluntary basis and were able to withdraw from the study at any stage. Informed consent was provided to all participants. The analyses were conducted anonymously.

### 2.2. Measures

#### 2.2.1. Attachment

The Attachment Styles Questionnaire [27] is based on Bowlby’s and Ainsworth’s theory of attachment [28]. It assesses three attachment styles: secure, avoidant, and anxious. The questionnaire consists of 24 items rated on a 7-point scale (from 1 = strongly disagree to 7 = strongly agree). The individual statements refer to emotions and feelings that accompany a person in a close marriage/partner relationship. Higher scores on each subscale indicate a greater intensity of the attachment styles reflected in a person’s behavior. The reliability coefficients for the current study were *α* = 0.87 (secure style), *α* = 0.81 (avoidant style), and *α* = 0.83 (anxious style).

#### 2.2.2. Sense of Coherence

The Sense of Coherence Scale (SOC-29) [29] assesses the intensity of sense of coherence, understood as the degree to which people have a relatively stable and active sense of certainty about their existence. The scale measures the three main components of sense of coherence: comprehensibility, manageability, and meaningfulness. The scale includes 29 items rated on a 7-point scale (from 1  =  never to 7  =  always). Higher scores represent a greater sense of coherence, which refers to the extent to which people perceive their life as comprehensible, manageable, and meaningful. The overall score is the arithmetic mean of the subscale scores. The reliability coefficients for the current study were *α* = 0.76 (comprehensibility), *α* = 0.80 (manageability), *α* = 0.83 (meaningfulness), and *α* = 0.86 (total score). 

#### 2.2.3. Illness Acceptance

The Acceptance of Life with the Disease Scale [30] assesses adaptation to illness, understood as an individual’s ability to accept health conditions in the context of their own illness. The scale consists of 20 items rated on a 5-point scale (from 1  =  never to 5  =  always). It has three subscales: satisfaction with life, reconciliation with the disease, and self-distancing from the disease. Their arithmetic mean gives an overall score, which was the only one used in this study. Higher scores reflect a higher level of illness acceptance. The reliability coefficient for the current study was *α* = 0.88.

### 2.3. Statistical Analyses

Data analyses were conducted with SPSS Statistics 25 and SPSS Amos 25. First, using the recommendations proposed by Soper [31] and Westland [32], and taking into account previous research findings [14,24], we conducted a power test calculation to establish a required sample size. A sample size of 284 participants (142 dyads) was deemed sufficient to detect a minimum effect size (*α* = 0.05, 1 − *β* = 0.80; estimated beta for actor and partner effects = 0.20). Our study sample consisted of 290 participants (145 women with breast cancer and their 145 male spouses). Second, two-tailed Pearson correlations were computed to both examine associations among all the variables and control the dyadic nonindependence. Third, the actor–partner interdependence model (APIM) was applied to determine both actor–actor and actor–partner effects for patients and their spouses. The actor–actor effect examines the associations between each dyad member’s own attachment and illness acceptance, whereas the actor–partner effect assesses the associations between each dyad member’s own attachment and the other member’s illness acceptance. Therefore, in the present study the actor–actor effect defined the relationship of the personal predictor variable (i.e., attachment) with the personal dependent variable (i.e., illness acceptance), while the actor–partner effect specified the relationship of the personal predictor variable with the partner’s dependent variable. To avoid confusion of terms, “partner” is used only to refer to the APIM partner effect and “spouse” to refer to the patient’s life partner.

The following requirements were checked before conducting path analysis: (a) Harman’s one-factor test was used to exclude common method variance and subsequent biases (as all items formed 18 distinct factors, with the first unrotated factor explaining only 20.78% of the variance, common method error was not present in this study); (b) the level of multicollinearity was acceptable, as the Variance Inflation Factor (VIF) was 1.29 for all the predictors. 

Finally, path analysis was used to test the APIM with the bootstrapping of 5000 samples and 95% confidence intervals (CIs) aimed at examining the direct and indirect effects [33]. The intended mediation analysis examined attachment as an independent variable; sense of coherence as a mediator; and illness acceptance as a dependent variable. All models were adjusted for age and education due to the following reasons: first, subjects’ age in SEM analysis may affect the relationships between variables; second, the age of cancer patients may determine perceptions of illness and attitudes toward illness; and third, respondents’ education may impinge on the level of comprehension of items and insight into their mental processes, which could influence the responses.

## 3. Results

### 3.1. Descriptive Statistics and Correlational Findings

The mean age of patients was 51.6 (SD = 10.59) years, and of spouses 53.2 (SD = 11.05) years. The mean tenure of relationship was 21.7 (SD = 11.27) years, with a range from 1.2 years to 51.5 years. Regarding marital status, 85.5% of the couples were married and 14.5% were cohabiting. Most participants had full-time/part-time work (59.2%), 25.8% were retired, and 15.0% were unemployed/at home. The mean time since cancer diagnosis was 2.81 (SD = 1.19) years.

Within the sociodemographic variables, only age was positively associated with comprehensibility (*r* = 0.15, *p* < 0.01) and illness acceptance (*r* = 0.15, *p* < 0.01). The duration of illness was negatively correlated with manageability (*r* = −0.21, *p* < 0.01), meaningfulness (*r* = 0.14, *p* < 0.05), and overall sense of coherence (*r* = 0.13, *p* < 0.05). No bivariate correlations between gender and the duration of illness and the attachment measures were statistically significant.

Further correlational findings among key study variables are summarized in Table 1. Patients’ secure attachment was positively correlated with their own comprehensibility, manageability, meaningfulness, overall sense of coherence, and illness acceptance. In contrast, patients’ avoidant and anxious attachment styles were negatively correlated with their own comprehensibility, manageability, meaningfulness, overall sense of coherence, and illness acceptance. The components of sense of coherence and its overall score from patients were positively associated with patients’ illness acceptance. 

For the spouses, secure attachment was positively correlated with comprehensibility, manageability, meaningfulness, overall sense of coherence, and illness acceptance. Spouses’ avoidant and anxious attachment styles were negatively correlated with comprehensibility, manageability, meaningfulness, overall sense of coherence, and illness acceptance. Finally, spouses’ sense of coherence and its components were positively related to their own illness acceptance. 

### 3.2. Testing Actor–Partner Interdependence Models: Path Analysis

#### 3.2.1. Secure Attachment

**Figure 1:**
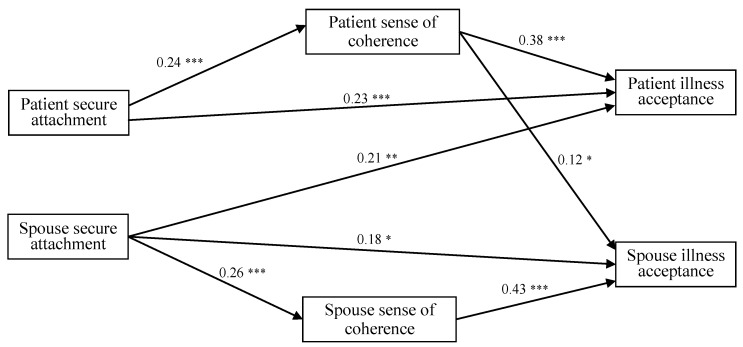
The final actor–partner interdependence model: anxious attachment, sense of coherence, and illness acceptance (standardized coefficients). * *p* < 0.05; ** *p* < 0.01; *** *p* < 0.001.

Path analysis was conducted to test the actor–partner interdependence model for secure attachment. The initial model had an unsatisfactory fit to the data: *χ*2 (3, *n* = 290) = 34.06; *p* < 0.001; NFI = 0.79; CFI = 0.78; RMSEA = 0.27; SRMR = 0.11. Although the χ^2^ value in the initial model was statistically significant, the other indices (i.e., NFI, CFI, RMSEA, and SRMR) did not reach a sufficient level of statistical significance. In addition, some of the direct paths between variables were nonsignificant. The model was then re-tested, considering path coefficients and modification indices to improve fit. This resulted in the final model, which showed a satisfactory fit to the data: *χ*2 (5, *n* = 290) = 9.24; *p* < 0.001; NFI = 0.94; CFI = 0.97; RMSEA = 0.07; SRMR = 0.06 (Figure 1). All the paths were statistically significant. When compared, the final model showed a better fit than the initial one: Δ*χ*2 (2) = 24.82, *p* < 0.001.

Following the principles of the actor–partner interdependence model, actor and partner effects were then analyzed. The associations between each dyad member’s own attachment and illness acceptance were identified as “actor–actor effects,” whereas the associations between each dyad member’s own attachment and the other member’s illness acceptance were identified as “actor–partner effects.” 

Within the actor–actor effects, we found two significant direct paths: from the patient’s secure attachment to the patient’s illness acceptance (E = 0.23, CI = 0.12, 0.33), and from the spouse’s secure attachment to the spouse’s illness acceptance (E = 0.18, CI = 0.05, 0.30). They both had positive associations. Within the actor–partner effects, there was only one direct path: from the patient’s secure attachment to the spouse’s illness acceptance (E = 0.21, CI = 0.08, 0.34). This also had a positive association. 

Next, the mediational relations for the actor–actor and actor–partner effects were calculated separately (Table 2), providing accurate values for different paths within the model. For the actor–actor effects, the patient’s sense of coherence mediated the relationship between the patient’s secure attachment and their own illness acceptance (E = 0.09, CI = 0.04, 0.16), and the spouse’s sense of coherence mediated the relationship between the spouse’s secure attachment and their own illness acceptance (E = 0.11, CI = 0.05, 0.19). Thus, both patients and spouses with higher secure attachment had a higher sense of coherence, which in turn was associated with their own higher illness acceptance, respectively. For the actor–partner effects, only the patient’s sense of coherence mediated the relationship between the patient’s secure attachment and the spouse’s illness acceptance (E = 0.03, CI = 0.01, 0.06). Patients with a more secure attachment had a higher sense of coherence, which in turn was associated with their spouse’s higher level of illness acceptance.

#### 3.2.2. Avoidant Attachment

**Figure 2:**
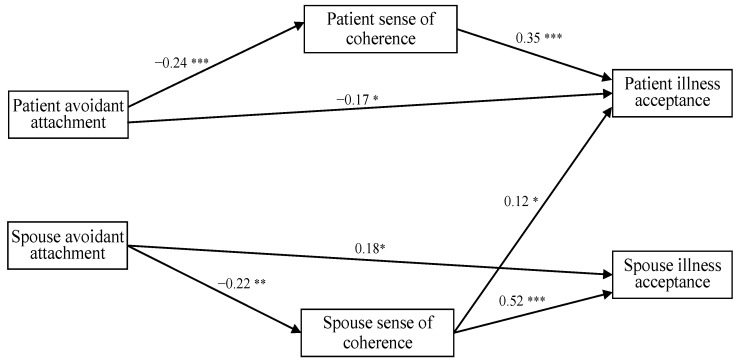
The final actor–partner interdependence model: avoidant attachment, sense of coherence, and illness acceptance (standardized coefficients). * *p* < 0.05; ** *p* < 0.01; *** *p* < 0.001.

A similar path analysis was conducted to test the APIM for avoidant attachment. The initial model did not satisfactorily fit the data: *χ*2 (3, *n* = 290) = 48.15; *p* < 0.001; NFI = 0.69; CFI = 0.68; RMSEA = 0.32; SRMR = 0.13. Despite the statistically significant χ^2^ value of the initial model, the other fit indices (i.e., NFI, CFI, RMSEA, and SRMR) were unsatisfactory. Some of the paths between variables were also nonsignificant. After re-testing the model and implementing modification indices to optimize fit, the final model was obtained, with a satisfactory fit to the data: *χ*2 (7, *n* = 290) = 13.23; *p* < 0.001; NFI = 0.92; CFI = 0.95; RMSEA = 0.07; SRMR = 0.07 (Figure 2). All the paths were statistically significant. When compared, the final model showed a better fit than the initial one: Δ*χ*2 (4) = 34.92, *p* < 0.001.

Based on the APIM, actor–actor and actor–partner effects were then examined for avoidant attachment. Within the actor–actor effects, only one significant direct path emerged: from the patient’s avoidant attachment to the patient’s illness acceptance (E = −0.17, CI = −0.27, −0.09). A higher patient’s avoidant attachment was associated with their own lower illness acceptance. However, no significant direct partner effect emerged in this model.

Then, we separately examined the mediational relations for actor–actor and actor–partner effects (Table 2). For the actor–actor effects, the relationship between the patient’s avoidant attachment and their own illness acceptance was mediated by their sense of coherence (E = −0.09, CI = −0.15, −0.04), and the relationship between the spouse’s avoidant attachment and their own illness acceptance was mediated by the spouse’s sense of coherence (E = −0.10, CI = −0.20, −0.03). Therefore, both patients and spouses with higher avoidant attachment had a lower sense of coherence, which in turn was associated with their own lower illness acceptance, respectively. For the actor–partner effects, only one mediational relationship emerged: the spouse’s sense of coherence mediated between their avoidant attachment and the patient’s illness acceptance (E = −0.03, CI = −0.07, −0.01). Spouses with higher avoidant attachment had a lower sense of coherence, which in turn was associated with the patient’s lower level of illness acceptance.

#### 3.2.3. Anxious Attachment

**Figure 3:**
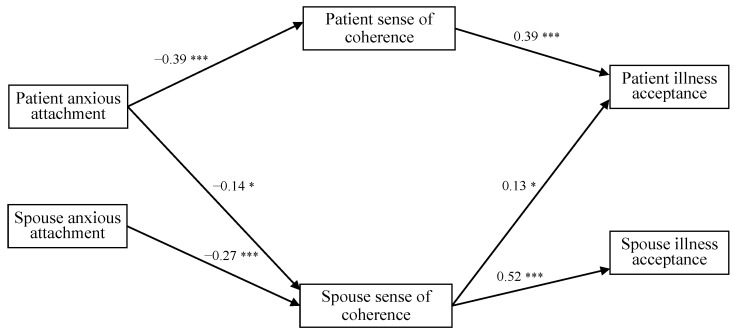
The final actor–partner interdependence model: anxious attachment, sense of coherence, and illness acceptance (standardized coefficients). * *p* < 0.05; *** *p* < 0.001.

Path analysis was also employed to test the model for anxious attachment. The initial model showed an unsatisfactory fit to the data: *χ*2 (3, *n* = 290) = 33.42; *p* < 0.001; NFI = 0.79; CFI = 0.79; RMSEA = 0.26; SRMR = 0.12. Though the χ^2^ value in the initial model was statistically significant, the other indices (i.e., NFI, CFI, RMSEA, and SRMR) did not have the significance required. Moreover, some of the direct paths did not reach statistical significance. Considering path coefficients and modification indices, the model was then re-tested to improve fit, and the final model showed a good fit to the data—*χ*2 (7, *n* = 290) = 11.35; *p* < 0.001; NFI = 0.93; CFI = 0.97; RMSEA = 0.06; SRMR = 0.06 (Figure 1)—with all its paths significant (Figure 3). The final model showed a better fit in comparison with the initial one: Δ*χ*2 (2) = 22.07, *p* < 0.001.

The actor–actor and actor–partner effects were examined within the APIM. We observed no statistically significant direct paths between anxious attachment and illness acceptance, either for patients or for spouses. However, indirect paths were found to be significant (Table 2). For the actor–actor effects, the patient’s sense of coherence mediated the relationship between the patient’s anxious attachment and their own illness acceptance (E = −0.17, CI = −0.25, −0.10), whereas the spouse’s sense of coherence mediated the relationship between the partner’s anxious attachment and their own illness acceptance (E = −0.15, CI = −0.21, −0.11) and between the spouse’s anxious attachment and their own illness acceptance (E = −0.14, CI = −0.22, −0.07). Both patients and spouses with higher anxious attachment had a lower sense of coherence, which subsequently was related to their own lower illness acceptance, respectively. Regarding the actor–partner effects, two mediational effects turned out significant: The spouse’s sense of coherence mediated the relationship between the patient’s anxious attachment and the spouse’s illness acceptance (E = −0.07, CI = −0.14, −0.01) and between the spouse’s anxious attachment and the patient’s illness acceptance (E = −0.04, CI = −0.08, −0.01), indicating that patients more anxiously attached had a lower sense of coherence, in turn related to their spouse’s lower levels of illness acceptance. Similarly, spouses more anxiously attached had a lower sense of coherence, in turn related to the patient’s lower levels of illness acceptance. The summarized results of the mediational actor–actor and actor–partner effects appear in Table 2, which provides accurate statistical values (i.e., estimates and confidence intervals) for different model paths.

## 4. Discussion

The current study examined the associations between attachment, sense of coherence, and illness acceptance in women with breast cancer and their spouses within a dyadic approach. To our knowledge, these findings are the first to demonstrate the complexity of the actor–partner interdependence relations in those groups. In general, our results support the effectiveness of a dyadic approach to investigating mediational relations in couples coping with cancer.

### 4.1. Associations Among Attachment, Sense of Coherence, and Illness Acceptance

For both patients and spouses, a more secure attachment style was positively associated with their sense of coherence and acceptance of cancer, whereas having avoidant and anxious attachment styles was negatively associated with these variables. This supports hypothesized associations between attachment, sense of coherence, and illness acceptance addressed by our first hypothesis. These findings are in line with previous studies which documented the association between a couple’s attachment styles, coherence-based personal resources, and their ability to adapt to illness [6,8,15]. The mechanism responsible for this may lie in the tendency to experience specific stress patterns that result from the emotional connection formed with their loved ones (i.e., parents, spouses).

From an attachment perspective, a serious and chronic disease like breast cancer is highly likely to induce intense stress, in which having a secure attachment style leads to a greater understanding of the different aspects of the illness, stronger meaning-related resourcefulness, and the ability to accept the negative consequences of the illness [10,34]. Conversely, anxiously and avoidantly attached patients and spouses may experience more stress, leading to greater difficulties in effectively using personal resources related to comprehensibility and meaningfulness, as well as show an inability to accept their illness due to attachment insecurity. Poorly managed emotional responses to stress [35] in turn are likely to relate to indices of poorer well-being, including difficulties in accepting a severe illness like cancer [36].

Furthermore, our SEM analysis of interpersonal relationships between attachment, sense of coherence, and illness acceptance revealed surprisingly interesting results. Among both patients and spouses, secure attachment was positively and anxious attachment was negatively associated with the other partner’s illness acceptance (directly or indirectly through sense of coherence). Thus, patients and spouses high in empathetic and safe attachment but low in worried and uncertain attachment had partners who reported significantly better illness acceptance. In cases of avoidant attachment, only patients reported better acceptance of the illness with lower levels of the other partner’s avoidant–dismissive behavior. Lower interpersonal effects from avoidant attachment may stem from the propensity of avoidantly attached individuals to minimize their own fears and anxieties in threatening contexts [4,5]. As a consequence, avoidantly attached spouses may emotionally distance themselves from their partner, which may limit their ability to accept the other person’s illness.

To our knowledge, these findings for the first time document this actor–partner association not only in cancer patients, but also in their partners. These findings also broaden our knowledge of dyadic approaches to examining couples coping with cancer by highlighting the importance of emotional communication and intimate relationships in understanding the specifics of cancer acceptance [37]. The level of acceptance of the disease will therefore depend on the interaction of secure versus insecure attachment between partners, which involves seeking closeness to others and disclosing one’s feelings for support and reassurance.

### 4.2. Actor–Actor Effects

Our second hypothesis assumed that a person’s sense of coherence would mediate the effect of their own attachment on their own illness acceptance (i.e., the actor–actor effect). Our results fully confirmed this assumption; for the patients and spouses, respectively, sense of coherence was found to mediate the association of secure, avoidant, and anxious attachment styles with illness acceptance. However, the nature of these relationships varied for different attachment styles. Secure attachment was related to a higher sense of coherence, which in turn was related to better illness acceptance. Conversely, avoidant and anxious attachment was associated with a lower sense of coherence, in turn associated with poorer illness acceptance. These findings are consistent with previous studies that showed the mediating function of sense of coherence in the relationship of attachment and college challenges with depressive symptoms in college students [24,26]. Yet, our study broadens this research by showing that sense of coherence can be an important mediating factor not only in non-clinical groups, but also in clinical groups of breast cancer patients and their spouses.

In the context of such a serious and life-threatening illness like cancer, individuals ideally seek stability, understanding, and meaning, which allow them to be more resilient to the illness, maintain their well-being, and improve their health [19,38]. A firm and well-established sense of coherence represents an underlying personal resource that enables effective coping strategies and the psychological overcoming of the negative consequences of cancer. This was the case for both our patients and their spouses. This interpretation is supported by the salutogenic model, which views sense of coherence as a general orientation that conveys a feeling of confidence in the face of illness, predictability about the availability of resources to manage challenges, and meaningfulness in coping with the hardships of illness [20,25]. Consequently, awareness of having a coherence-based resource allows patients and their spouses to make more effective use of the quality and extent of their attachment and marital emotional support.

### 4.3. Actor–Partner Effects

In examining the interpersonal effects of sense of coherence in the link between attachment and illness acceptance, we found significant associations for patients and spouses, which also confirm the third hypothesis that assumed a mediational effect of a person’s sense of coherence between their own attachment and the illness acceptance of their partner (i.e., the actor–partner effect). Specifically, patients high in secure attachment and low in anxious attachment were characterized by a higher sense of coherence, in turn associated with better acceptance of the illness by spouses. Correspondingly, spouses with high avoidant and anxious attachment had a lower sense of coherence, in turn related to poorer patient acceptance of the illness.

This is an interesting result in the context of the recent literature on attachment and illness acceptance in couples coping with cancer, which points to a cross-over or interdependent approach to adjustment to cancer that is expressed in a simultaneous examination of one’s own attitudes toward one’s spouse’s illness [15,22]. Thus, forming a personal disposition of comprehensibility, manageability, and meaningfulness (i.e., the core components of coherence) may be motivated by the patient’s need to manage personal cancer-related distress, which overall brings significant benefits to the spouse. Conversely, developing a sense of coherence is likely to be driven by the spouse’s need to cope with the personal stress of cancer, which overall is beneficial for the patient. This view is well grounded in the inter-relational approach to experiencing illness, an approach which emphasizes the interdependence in how spouses, or even family members more broadly, experience the effects of illness [8,9,39]. Showing an emotional, secure attachment and avoiding anxious expressions to partners appears to depend on one’s sense of coherence, which helps to reduce the other person’s distress and increase efforts to accept the disease. This is consistent with George-Levi et al.’s observation that expressing affection to partners may be motivated by empathetic concern or personal egocentrism in order to reduce one’s own distress [40].

Finally, an unexpected result was the lack of association between attachment and illness acceptance for a secure attachment style in spouses and an avoidant attachment style in patients. These findings partly contrast with Ramos et al. [8], where a patient’s avoidant attachment was related to a spouse’s physical well-being through communication styles (i.e., disclosure and holding back). First, perhaps our study investigated illness acceptance instead of physical well-being, which are rather different conceptual constructs from a psychological perspective; second, as a mediator, the current study used sense of coherence instead of communication styles, which may have determined the function of couples’ motives to assess the illness and the context in which the assessment occurred. A recent study supports this interpretation: among couples struggling with cancer, the relationships between attachment and measures of adjustment to illness were highly complex and depended on the specific constructs used in a particular study [10].

### 4.4. Study Limitations

While this study has provided sound scientific effects, it has its limitations. First, due to the specific sociodemographic characteristics of the country where the study was conducted, the sample mostly comprised Caucasians who were economically well-off and in long-term relationships, factors which at least preclude our results’ generalizability to more culturally diverse groups. Furthermore, the measures used for the tested variables were self-reports, creating a potential risk of subjective bias. Future research could employ other types of measures that generate more objective data (e.g., reaction time measurement or physiological responses to illness). Additionally, our single-point measurement strategy precluded observing changes over time, limiting the possibility to discuss causation. Finally, we did not examine the interaction of patient and spouse attachment dimensions, which could influence the mediational role of sense of coherence or the relationship of attachment with illness acceptance.

## 5. Conclusions

Overall, our study has made a significant contribution to the existing literature by providing dyadic analyses on sense of coherence mediating between attachment and illness acceptance in couples coping with a serious illness (i.e., breast cancer). A higher use of secure attachment and lower use of insecure attachment in conjunction with the mediation of sense of coherence were associated with better illness acceptance in couples facing breast cancer. We also showed that accounting for all attachment styles, the mediational values of the actor–actor and actor–partner models were different: the former were more frequent than the latter. Ultimately, these results can hopefully lead to the development of intervention programs that benefit couples’ congruence to minimize the negative consequences of cancer and maintain well-being.

## Figures and Tables

**Table 1:** Correlations among attachment styles, sense of coherence, and illness acceptance among patients and spouses.

Variables	1.	2.	3.	4.	5.	6.	7.	8.
1. Secure attachment	–	−0.53 ***	−0.44 ***	0.18 *	0.24 **	0.20 **	0.24 **	0.37 ***
2. Avoidant attachment	−0.40 ***	–	0.48 ***	−0.19 *	−0.23 **	−0.23 **	−0.24 **	−0.30 ***
3. Anxious attachment	−0.39 ***	0.44 ***	–	−0.35 ***	−0.37 ***	−0.29 ***	−0.39 ***	−0.30 ***
4. Comprehensibility	0.25 ***	−0.15	−0.29 ***	–	0.68 ***	0.49 ***	0.86 ***	0.35 ***
5. Manageability	0.19 *	−0.25 ***	−0.32 ***	0.70 ***	–	0.73 ***	0.89 ***	0.41 ***
6. Meaningfulness	0.25 ***	−0.19 *	−0.16 *	0.56 ***	0.63 ***	–	0.82 ***	0.44 ***
7. Sense of coherence	0.26 ***	−0.22 **	−0.30 ***	0.89 ***	0.89 ***	0.80 ***	−	0.45 ***
8. Illness acceptance	0.30 ***	−0.19 *	−0.17 *	0.40 ***	0.47 ***	0.51 **	0.52 ***	−
Patients’ mean (SD)	6.00 (0.86)	1.79 (0.89)	2.69 (1.06)	4.39 (0.81)	5.24 (0.81)	5.56 (0.83)	5.01 (0.71)	3.42 (0.66)
Spouses’ mean (SD)	6.14 (0.67)	1.67 (0.81)	2.83 (1.05)	4.23 (0.89)	5.04 (0.82)	5.64 (0.76)	4.90 (0.73)	3.53 (0.60)

Note: Above the diagonal represents correlations for patients; below the diagonal represents correlations for spouses. * *p* < 0.05; ** *p* < 0.01; *** *p* < 0.001.

**Table 2:** Mediational effects: bootstrapped standardized estimates and 95% confidence intervals (CIs) from actor–partner interdependence modeling for patients (PTs) and spouses (SPs).

Effect Type	Model Pathways	Estimate	95% CI
Actor–actor effects Actor–partner effects	PT secure attachment → PT sense of coherence → PT illness acceptance	0.09	(0.04, 0.16)
	PT avoidant attachment → PT sense of coherence → PT illness acceptance	−0.09	(−0.15, −0.04)
	PT anxious attachment → PT sense of coherence → PT illness acceptance	−0.17	(−0.25, −0.10)
	PT anxious attachment → SP sense of coherence → PT illness acceptance	−0.15	(−0.21, −0.11)
	SP secure attachment → SP sense of coherence → SP illness acceptance	0.11	(0.05, 0.19)
	SP avoidant attachment → SP sense of coherence → SP illness acceptance	−0.11	(−0.20, −0.03)
	SP anxious attachment → SP sense of coherence → SP illness acceptance	−0.14	(−0.22, −0.07)
	PT secure attachment → PT sense of coherence → SP illness acceptance	0.03	(0.01, 0.07)
	SP avoidant attachment → SP sense of coherence → PT illness acceptance	−0.03	(−0.07, −0.01)
	PT anxious attachment → SP sense of coherence → SP illness acceptance	−0.07	(−0.14, −0.01)
	SP anxious attachment → SP sense of coherence → PT illness acceptance	−0.04	(−0.08, −0.01)

Abbreviations: patient—PT; spouse—SP.

## Data Availability

The data presented in this study are available at OSF Home: https://osf.io/kvqmn. (accessed on 27 September 2024).

## References

[1] Sedeta E.T., Jobre B., Avezbakiyev B. (2023). Breast cancer: Global patterns of incidence, mortality, and trends. J. Clin. Oncol..

[2] The Incidence and Mortality of Female Cancers in Poland Are Increasing. https://www.onkonet.pl/n_n_nowotwory_kobiece_potrzeba_zmian.php.

[3] Bradford A.B., Drean L., Sandberg J.G., Johnson L.N. (2020). They may disapprove, but I still love you: Attachment behaviors moderate the effect of social disapproval on marital relationship quality. Fam. Process.

[4] Bowlby J. (1982). Attachment and Loss: Vol. 1. Attachment.

[5] Bowlby J. (1988). A Secure Base: Parent-Child Attachment and Healthy Human Development.

[6] Mikulincer M., Shaver P.R. (2019). Attachment orientations and emotion regulation. Curr. Opin. Psychol..

[7] Milbury K., Yang C., Liao Z.X., Tsao A.S., Bruera E. (2018). Relationship processes and symptom burden in couples coping with metastatic lung cancer. J. Clin. Oncol..

[8] Ramos K., Langer S.L., Todd M., Romano J.M., Ghosh N., Keefe F.J., Baucom D.H., Syrjala K.L., Porter L.S. (2020). Attachment style, partner communication, and physical well-being among couples coping with cancer. Pers. Relatsh..

[9] Ramos K., Leo K., Porter L.S., Romano J.M., Baucom B.R., Langer S.L. (2023). Attachment in couples coping with cancer: Associations with observed communication and long-term health. Int. J. Environ. Res. Public Health.

[10] Karveli S., Galanis P., Mitropoulou E.M., Karademas E., Markopoulos C. (2023). The role of attachment styles on quality of life and distress among early-stage female breast cancer patients: A systematic review. J. Clin. Psychol. Med. Settings.

[11] Nissen K.G. (2016). Correlates of self-rated attachment in patients with cancer and their caregivers: A systematic review and meta-analysis. Psychooncology.

[12] Xiaoyun C., Fenglan L. (2020). The relationships among insecure attachment, social support and psychological experiences in family caregivers of cancer inpatients. Eur. J. Oncol. Nurs..

[13] Hiebler-Ragger M., Nausner L., Blaha A., Grimmer K., Korlath S., Mernyi M., Unterrainer H.F. (2021). The supervisory relationship from an attachment perspective: Connections to burnout and sense of coherence in health professionals. Clin. Psychol. Psychother..

[14] Shalev D., Jacobsen J.C., Rosenberg L.B., Brenner K.O., Seaton M., Jackson V.A., Greer J.A. (2022). (Don’t) Leave Me Alone: Attachment in Palliative Care. J. Palliat. Med..

[15] Crangle C.J., Torbit L.A., Ferguson S.E., Hart T.L. (2020). Dyadic coping mediates the effects of attachment on quality of life among couples facing ovarian cancer. J. Behav. Med..

[16] Trejnowska A., Goodall K., Rush R., Ellison M., McVittie C. (2020). The relationship between adult attachment and coping with brain tumour: The mediating role of social support. Psychooncology.

[17] Tao L., Lv J., Tan X., Hu X., Fu L., Li J. (2024). Relationships between attachment style, emotional self-disclosure, and quality of life among young breast cancer survivors: A cross-sectional study. Semin. Oncol. Nurs..

[18] Krok D., Zarzycka B., Telka E. (2021). The interplay of religious and nonreligious meaning-making on psychological well-being in gastrointestinal cancer patients. Int. J. Psychol. Relig..

[19] Hinz A., Schulte T., Ernst J., Mehnert-Theuerkauf A., Finck C., Wondie Y., Ernst M. (2023). Sense of coherence, resilience, and habitual optimism in cancer patients. Int. J. Clin. Health Psychol..

[20] Antonovsky A. (1979). Health, Stress and Coping.

[21] Eriksson M., Mittelmark M.B., Bauer G.F., Vaandrager L., Pelikan J.M., Sagy S., Eriksson M., Lindström B., Magistretti C.M. (2022). The sense of coherence: The concept and its relationship to health. The Handbook of Salutogenesis.

[22] Pasek M., Dębska G., Wojtyna E. (2017). Perceived social support and the sense of coherence in patient–caregiver dyad versus acceptance of illness in cancer patients. J. Clin. Nurs..

[23] Krok D., Telka E. (2022). Spousal support and illness acceptance in breast cancer patients: The mediating function of meaning in life and sense of coherence. Fam. Forum.

[24] Ying Y.W., Lee P.A., Tsai J.L. (2007). Attachment, sense of coherence, and mental health among Chinese American college students: Variation by migration status. Int. J. Intercult. Relat..

[25] Antonovksy A. (1987). Unraveling the Mystery of Health: How People Manage Stress and Stay Well.

[26] Ying Y.W., Lee P.A., Tsai J.L. (2007). Predictors of depressive symptoms in Chinese American college students: Parent and peer attachment, college challenges and sense of coherence. Am. J. Orthopsychiatry.

[27] Plopa M. (2008). Kwestionariusz Stylów Przywiązaniowych (KSP) [The Attachment Styles Questionnaire].

[28] Ainsworth M.D.S. (1978). The Bowlby-Ainsworth attachment theory. Behav. Brain Sci..

[29] Antonovsky A. (1993). The structure and properties of the sense of coherence scale. Soc. Sci. Med..

[30] Janowski K., Steuden S., Pietrzak A., Krasowska D., Kaczmarek Ł., Gradus I., Chodorowska G. (2012). Social support and adaptation to the disease in men and women with psoriasis. Arch. Dermatol. Res..

[31] Soper D.S. A-Priori Sample Size Calculator for Structural Equation Models. https://www.danielsoper.com/statcalc/calculator.aspx?id=89.

[32] Westland J.C. (2010). Lower bounds on sample size in structural equation modeling. Electron. Commer. Res. Appl..

[33] Hayes A.F., Preacher K.J., Hancock G.R., Mueller R.O. (2013). Conditional process modeling: Using structural equation modeling to examine contingent causal processes. Structural Equation Modeling: A Second Course.

[34] Cassidy T., McLaughlin M. (2024). Caring for a child with cancer: The role of attachment, self-compassion and social support. Child Care Pract..

[35] Snyder K.S., Luchner A.F., Tantleff-Dunn S. (2024). Adverse childhood experiences and insecure attachment: The indirect effects of dissociation and emotion regulation difficulties. Psychol. Trauma.

[36] Krok D., Telka E., Kocur D. (2024). Perceived and received social support and illness acceptance among breast cancer patients: The serial mediation of meaning-making and fear of recurrence. Ann. Behav. Med..

[37] Leo K., Langer S.L., Porter L.S., Ramos K., Romano J.M., Baucom D.H., Baucom B.R. (2024). Couples communication and cancer: Sequences and trajectories of behavioral affective processes in relation to intimacy. J. Fam. Psychol..

[38] Asaba K., Okawa A. (2021). Moderating effect of sense of coherence on the relationship between symptom distress and health-related quality of life in patients receiving cancer chemotherapy. Support Care Cancer.

[39] Chen M., Gong J., Cao Q., Luo X., Li J., Li Q. (2021). A literature review of the relationship between dyadic coping and dyadic outcomes in cancer couples. Eur. J. Oncol. Nurs..

[40] George-Levi S., Vilchinsky N., Tolmacz R., Khaskiaa A., Mosseri M., Hod H. (2016). “It takes two to take”: Caregiving style, relational entitlement, and medication adherence. J. Fam. Psychol..

